# Sex differences in Parkinson’s disease

**DOI:** 10.1016/j.yfrne.2014.02.002

**Published:** 2014-08

**Authors:** Glenda E. Gillies, Ilse S. Pienaar, Shiv Vohra, Zahi Qamhawi

**Affiliations:** Division of Brain Sciences, Department of Medicine, Imperial College London, Hammersmith Campus, Du Cane Road, London W12 0NN, United Kingdom

**Keywords:** Sex differences, Parkinson’s disease, Nigrostriatal dopaminergic pathways, Sex hormones, Neuroprotection

## Abstract

Parkinson’s disease (PD) displays a greater prevalence and earlier age at onset in men. This review addresses the concept that sex differences in PD are determined, largely, by biological sex differences in the NSDA system which, in turn, arise from hormonal, genetic and environmental influences. Current therapies for PD rely on dopamine replacement strategies to treat symptoms, and there is an urgent, unmet need for disease modifying agents. As a significant degree of neuroprotection against the early stages of clinical or experimental PD is seen, respectively, in human and rodent females compared with males, a better understanding of brain sex dimorphisms in the intact and injured NSDA system will shed light on mechanisms which have the potential to delay, or even halt, the progression of PD. Available evidence suggests that sex-specific, hormone-based therapeutic agents hold particular promise for developing treatments with optimal efficacy in men and women.

## Highlights

• Biological sex differences are found in the nigrostriatal dopaminergic pathway.
• These may underpin the greater male susceptibility to develop Parkinson’s disease.
• Female gonadal factors (estradiol) provide resilience to dopamine loss.
• Male gonadal factors (testosterone/estradiol) fail to protect/worsen dopamine loss.
• Sex-specific hormone-based therapies hold promise for novel treatments.

## 1 Introduction

One’s sex is increasingly recognised as a factor which influences the incidence and/or nature of all major complex diseases, including neurodegenerative and neuropsychiatric disorders. This, in turn, may be determined by biological sex differences in brain organisation, structure and function, which are determined genetically and epigenetically (Gabory et al., 2009; Kaminsky et al., 2006; McCarthy et al., 2009; Cahill, 2006). This review will consider these phenomena in relation to Parkinson’s disease (PD), numerous aspects of which strongly support the urgent need for a better understanding of brain sex dimorphisms in the intact and injured brain, in order to design improved therapies with optimal efficacy in male and female patients alike.

## 2 Parkinson’s disease: differences between men and women

### 2.1 Aetiology and pathology of PD

PD is the second most common neurodegenerative disorder, affecting approximately 0.3% of people in the developed world. This rises rapidly to 3% for individuals over the age of 65 years, to demonstrate that advanced age comprises a major risk factor (Dexter and Jenner, 2013). In addition, such figures highlight the increasing burden that treatment of PD place on health care systems worldwide, as the population life-expectancy increases in several countries. Clinically, PD is a movement disorder that is characterised by motor symptoms such as bradykinesia with rigidity, tremor at rest, gait disturbances and difficulty in swallowing and producing speech. Non-motor symptoms associated with the disorder include anxiety, depression, insomnia, dementia, autonomic dysfunction and constipation, which can often reduce patients’ quality of life even more significantly than motor aspects (Weintraub et al., 2008; Jenner et al., 2013). A major pathological lesion associated with PD is the loss of midbrain dopaminergic (DAergic) neurons in the substantia nigra pars compacta (SNc) and the consequent loss of DA input to the caudate nucleus and putamen (the striatum). This nigrostriatal DA (NSDA) pathway plays a central role in regulating fine motor control, and its degeneration thus leads to the primary motor symptoms of PD. In up to approximately 10% of cases, rare familial genetic mutations have been identified as causing PD. However the vast majority of cases are of unknown cause and are termed idiopathic or sporadic (Klein and Schlossmacher, 2007). Yet, studies have begun to cast light on the cellular and molecular processes which may underlie the degeneration of the NSDA system. Putative pathological substrates include but are not limited to mitochondrial dysfunction (Vives-Bauza et al., 2010; Pienaar and Chinnery, 2013) accompanied by the excessive production of radical oxygen species (Mattson, 2006), the formation of protein aggregates, termed Lewy bodies(principally composing of α-synuclein and ubiquitin) within the surviving DAergic neurons and microglial inflammation (Halliday and Stevens, 2011). Collectively, these observations support the general concept that PD is a complex disease, representing a clinical syndrome with an aetiology that is likely to comprise of interactions between multiple genetic factors, the environment, the immune system and aging (Jenner et al., 2013; Klein and Schlossmacher, 2007).

### 2.2 Sex differences in PD

After aging, epidemiological studies have revealed the male sex as a prominent risk factor for developing PD at all ages and for all nationalities studied. Reports of male to female ratios for incidence rates vary from 1.37 to 3.7 (Baldereschi et al., 2000; Swerdlow et al., 2001; Van Den Eeden et al., 2003; Wooten et al., 2004; Shulman and Bhat, 2006; Taylor et al., 2007), with a large meta-analysis study suggesting that, in any specific time-frame, twice as many men than women suffer from PD (Elbaz et al., 2002).

#### 2.2.1 Clinical profile of PD

In addition to differences in its prevalence in men and women, many studies have reported sex differences in the clinical profile of PD. For example, some studies report that the age of onset of PD is approximately 2 years later in women compared with men (Haaxma et al., 2007; Alves et al., 2009). Although earlier work contradicted this finding (Baba et al., 2005), such discrepancies can be explained, at least in part, by sex differences in disease presentation. For example, several studies suggest that females present with a milder PD phenotype, which is most notable in the early clinical stages, especially prior to the introduction of anti-parkinsonian medication (Shulman and Bhat, 2006; Haaxma et al., 2007; Miller and Cronin-Golomb, 2011). Compared to men, women are also reported to present more often with tremor, a symptom that correlates both with a later age of onset and a slower rate of decline of motor impairment (Haaxma et al., 2007). Other symptoms that were found to be more prevalent in women than in men include nervousness, sadness, depression and constipation, whereas men suffered more from daytime sleepiness, dribbling and sex-related symptoms (Martinez-Martin and Falup, 2012). Rigidity and rapid eye movement behaviour disorder occurs more frequently in men, whereas women are more likely to have dyskinesias and PD-associated depression than men (Martinez-Martin and Falup, 2012). A sex-specific pattern is also emerging for PD-associated cognitive changes, with deficits in verbal fluency and recognition of facial emotions being more prevalent in men, whilst a reduction in visuospatial cognition occur more frequently in women (Miller and Cronin-Golomb, 2011). Additionally, the efficacy, tolerability and pharmacokinetics of drugs used for treating PD appear to differ in men versus women (Shulman and Bhat, 2006).

In support of the notion that sex differences in disease susceptibility may be determined, at least in part, by biological sex differences in various affected brain regions, a number of differences in motor and sensory functions, which rely on the NSDA system have been noted in healthy men and women. For example, in tests of fine motor control and speech articulation, women generally outperform men (Jennings et al., 1998). The advent of real-time *in vivo* imaging techniques also provides direct evidence for innate differences in NSDA transmission in men and women. These include differences in basal striatal DAergic neuron dynamics (Pohjalainen et al., 1998; Lavalaye et al., 2000; Kaasinen et al., 2001; Mozley et al., 2001; Laakso et al., 2002), amphetamine-stimulated DA release (Munro et al., 2006) and sex-related differences in the functional relationship between regional DA release and motor performance, affect and cognitive function (Mozley et al., 2001; Riccardi et al., 2011). Sex differences in the healthy NSDA system are further corroborated by evidence discussed below from gene profiling studies (Section 2.2.2) and the discovery that the *SRY* gene (sex determining region on the Y sex chromosome) is expressed in SNc DA neurons in humans (Section 2.3.1) as well as rodents (Section 6.1).

#### 2.2.2 Molecular pathology of PD

New technologies which enable dissection of the molecular pathology of PD are beginning to provide a more objective analysis of underlying sexual dimorphisms. For example, the SNc DAergic neurons are identifiable in post-mortem brains, due to their dark neuromelanin pigmentation. This uniquely enables single-cell laser capture micro-dissection of this neuronal population, and has been coupled with microarray analysis of DNA in order to investigate gene expression profiles obtained from post-mortem brains of control subjects with individuals who, in life, had been diagnosed with late-stage idiopathic PD (Simunovic et al., 2011; Cantuti-Castelvetri et al., 2007). In the normal brain, genes involved in signal transduction and neuronal maturation were up-regulated in women, whereas genes implicated in PD pathogenesis, when harbouring specific mutations (e.g. α-synuclein and PINK-1), were up-regulated in men. In the DAergic neurons surviving in PD-affected brains, changes in the expression of genes encoding for protein kinase activity and genes associated with proteolysis and Wnt signalling predominated in women, whereas predominant expressional changes for genes involved in protein- and copper-binding activities occurred in men (Simunovic et al., 2011; Cantuti-Castelvetri et al., 2007). These studies demonstrate that gene expression profiles in normal SNc DAergic neurons are sex-specific and suggest a bias in males which may underlie the predisposition to develop PD. They also indicate that adaptive processes in the surviving DAergic neurons proceed via different mechanisms in males and females, suggesting that the nature of the disease, and potentially the response to treatment, may be sex-specific.

Collectively, clinical and molecular studies clearly support the notion that women are relatively protected from PD compared with men. They also underscore the need for a better knowledge of the basis of sex differences in PD. Investigations into the pathophysiology underlying sex differences in the presentation, progression and treatment responses in PD are in their infancy, but offer considerable potential for improving clinical assessment and treatment of the disease.

### 2.3 Genetic and epigenetic factors contributing to sex bias in PD

The influence of genetic and epigenetic factors underlying disease is a vast subject, and this section will briefly focus only on areas of relevance to sex differences in PD. Environmental factors, which can alter the epigenetic signature, shall be considered, and in this context, sex and sex hormones, as well as stress and stress hormones, can be included as environmental factors since hormonal effects include DNA methylation and histone modifications, thereby altering epigenetic regulation of autosomal genes and potentially influencing differential susceptibility to complex diseases (Kaminsky et al., 2006).

#### 2.3.1 Genes

The genetics of PD is a rapidly growing field. To-date, mutations in at least 17 different genes have been identified as the cause of the rare familial forms of the disease (Dexter and Jenner, 2013). These genes often encode proteins that are associated with molecular pathways that are affected in sporadic forms of the disease. For example, mutations in the gene encoding α-synuclein accounts for only a very small proportion of familial PD. Yet protein aggregations containing α-synuclein (Lewy bodies) comprise a diagnostic pathology related to the final stages of DA neuronal loss in idiopathic PD and indicate altered protein aggregation as a contributory cause. Other mutations causing familial variants of PD involve genes that protect against mitochondrial dysfunction (PINK-1) and oxidative stress (DJ-1), all of which are pathological processes that have been implicated in idiopathic PD. However, to date there is no clear evidence to link these genes to sex differences in PD.

In view of the evidence that estrogen may be neuroprotective, a small number of studies have investigated the relationship between single nucleotide polymorphisms (SNPs) in estrogen-related genes and the onset and development of PD (see Sections 2.3.3 and 4). Although some authors have suggested a complex association, which may be more prevalent in one sex over the other (Palacios et al., 2011; Chung et al., 2011), no clear correlations have been reported to exist between SNPs in estrogen receptors (ERs) and the prevalence of PD. Some studies have shown, however, that genetic variation in ERβ was associated with an early-age (between ages 20 and 50 years) onset of PD (Westberg et al., 2004; Hakansson et al., 2005; Maraganore et al., 2002), which accounts for around 10% of all cases (Dexter and Jenner, 2013). However, it remains to be determined how SNPs influence disease risk (Klein and Schlossmacher, 2007). Such explorations generally require interrogation on a large scale, taking into account several environmental factors and demographic characteristics, as well as the difficulties inherent in detecting the small effects of common genetic variants in population-based, case-controlled studies. In this regard, as single-gene effects account for so few PD cases, other factors must account for sex differences.

Evidence in humans and other species (see Section 6.1) suggests that the sex chromosomes themselves are likely contributors to biological sex differences and could potentially influence sex bias in many common, complex diseases (Kaminsky et al., 2006). This could be due to a direct effect of Y chromosome genes (present only in males), or to incomplete silencing of X chromosome genes in XX-chromosome containing females, as well as sex differences in the genomic imprinting of X-chromosome genes (Federman, 2006; van Nas et al., 2009; Arnold and Burgoyne, 2004). One Y chromosome gene in particular, *SRY*, may be of relevance to NSDA function and, possibly also to PD. The actions of this gene were classically thought to be restricted to sex determination early in development due to its role in directing formation of the testes, but its expression during development and adulthood has now been identified in a number of male non-reproductive tissues, including the brain in humans (Ngun et al., 2011), rats and mice (see Section 6.1) (Dewing et al., 2006). Moreover, investigations done on human post-mortem brain specimens, revealed SRY immunoreactivity (IR) co-localised with a sub-population of SNc neurons that express tyrosine hydroxylase (TH). As TH is the rate-limiting step in DA synthesis, this identifies the SRY-IR neurons as belonging to the NSDA population (Czech et al., 2012). Using retinoic acid-induced differentiation of human male-derived precursor NT2 cells into DA cells, the same study confirmed co-localisation of SRY and TH. Furthermore, it was shown that SRY positively regulated expression of enzymes involved in the regulation of DA synthesis (TH, DOPA decarboxylase and dopamine β-hydroxylase) and metabolism (monoamine oxidase A) in the M17 cell line derived from a male neuroblastoma. These findings support the view that the male NSDA system is uniquely regulated by SRY. Future studies are needed to determine whether this contributes to the molecular and functional sex differences inherent to this pathway (see Section 2.2), and to elucidate on the role of SRY in the development of midbrain DA-related disorders that show a sex bias, such as PD.

#### 2.3.2 Life-style

Sex differences in life-style may also be contributory factors to the sex differences seen in PD, which could interact with a genetic predisposition (Wooten et al., 2004; Das et al., 2011), supporting the multiple-hit hypothesis for developing PD (Carvey et al., 2006). For example, exposure to herbicides (such as paraquat) has been associated with an increased risk for PD; as men were traditionally more likely to be agricultural workers, occupational exposure to agrichemicals could introduce differential risk exposure in men compared to women (Semchuk et al., 1992). Equally, head trauma has been linked to an increased risk for men to develop PD. In this regard, men show increased likelihood to suffer traumatic brain injury due to their greater exposure to events such as road traffic accidents or contact sports (Bruns and Hauser, 2003; Lehman et al., 2012).

Although there is not yet any conclusive evidence for a direct relationship, exposure to emotionally stressful events is another environmental factor which could increase the likelihood for developing PD. Stressful experiences compromise striatal DA release and motor function in healthy men and women (Cahill, 2006; Mozley et al., 2001), and can also exacerbate motor symptoms in PD patients (Macht et al., 2005). Additionally, poor strategies for coping with emotional stress may impact negatively on of life in PD (Whitworth et al., 2013), whilst an accumulation of stressful life events does appear to contribute to PD risk (Clark et al., 2013). Emerging evidence demonstrates that responses to, and strategies for coping with emotional or psychogenic stress differ notably in men (typified as the ‘fright, fight or flight’ response) and women (typified as the ‘tend and befriend’ response) (Klein and Corwin, 2002; Kudielka and Kirschbaum, 2005). Indeed, sex differences in terms of responses to stressful events are identifiable from functional magnetic resonance imaging (fMRI) data. In this regard, Wang and colleagues (2007) reported that men’s and women’s responses to a mild/moderate stressor could be distinguished to a very high accuracy based on real-time *in vivo* brain imaging alone (Wang et al., 2007). Additionally, biological differences between men and women have been noted as a major factor for consideration in the impact of stress on brain structure, function and pathology (Lupien et al., 2009). Taken together, underlying sex differences in stress-responsive circuitry are further contenders for contributing differential male/female risk factors for developing PD.

#### 2.3.3 Sex hormones

Sex hormones are by far the most important factors for driving structural and functional sexual differentiation in the brain, whilst also being critical drivers of sex differences in disease susceptibility. In studies focusing on such aspects, most attention has focused on estrogens, especially on 17β-estradiol (E2), the most abundant, naturally occurring estrogen in non-pregnant mammals, which is widely recognised to have neuroprotective actions and, therefore, may confer the advantage in diseases where women generally fare better, such as PD. The epidemiological and clinical evidence for E2 neuroprotection against PD in women has been the subject of a number of in-depth reviews (Shulman and Bhat, 2006; Dluzen and Horstink, 2003; Liu and Dluzen, 2007; Bourque et al., 2009), hence only the salient features will be summarised here. Women who underwent bilateral oophorectomy before menopause have an increased risk of developing PD (Benedetti et al., 2001; Ragonese et al., 2006; Rocca et al., 2008). At menses, when estrogen levels are lowest, PD symptom severity may worsen (Quinn and Marsden, 1986). Several reports also suggest that estrogen-based hormone replacement therapy can relieve PD symptoms when given in the early stages of the disease (Benedetti et al., 2001; Saunders-Pullman et al., 1999; Tsang et al., 2000) and decrease the risk of developing PD (Liu and Dluzen, 2007; Currie et al., 2004), whereas PD symptoms may deteriorate on cessation of therapy (Sandyk, 1989). However, there are some clinical reports, albeit in the minority, which fail to find evidence of estrogen neuroprotection (Popat et al., 2005). It should be borne in mind that the majority of clinical studies have been retrospective, and considerable variations in hormone replacement regimes, as well as the duration of the period of hypo-estrogenicity in the women studied are likely to represent confounding factors in interpretation of study data (Wise et al., 2005). Larger scale, prospective, controlled double-blind cross-over studies would be needed to reach firm conclusions and, thus far, any interaction between sex hormones and PD in men remains unexplored, although animal studies suggest potential detrimental effects of male gonadal factors (see Section 4). Notably, however, sex differences in PD may remain after menopause (Schrag et al., 2000), suggesting that estrogenic neuroprotection may be just one piece in the jig-saw puzzle, by which sex differences seen in PD may be explained.

In summary, the evidence discussed thus far favours the view that sex influences the normal functioning of the healthy NSDA pathway, as well as the nature and incidence of the degenerative processes affecting this pathway; sex hormones, specifically estrogens in females, seem to play a key role. Clinical studies are, however, limited in their ability to dissect the nature of estrogen’s actions on the brain, and there is no experiment of nature analogous to the menopause or menstrual cycle in women, to provide clues about potential hormonal influences in men. Therefore, pre-clinical experimental models of PD have an important role to play for filling these information gaps.

## 3 Sex differences in experimental Parkinson’s disease,

As the causes of PD are largely unknown, classical experimental *in vivo* and *in vitro* models of PD seek to mimic the prime pathological lesion associated with disease, namely NSDA degeneration using selective neurotoxins (Pienaar et al., 2012); more recently, genetic modifications of the genes identified as causing the rare familial forms of PD have also been recreated in animal models (Blesa et al., 2012). Although no single model perfectly recreates all aspects of the disease, they have helped elucidate much of what we know regarding the aetiology, pathology and molecular mechanisms of PD, whilst having been instrumental in developing new, and optimising existing treatments.

### 3.1 Animal models

The toxin-based models have been used widely for the study of sex differences and hormonal influences in PD. The most commonly used models involve central administration of 6-hydroxydopamine (6-OHDA) directly to the NSDA pathway, mainly in rodents (Dluzen, 1997; Murray et al., 2003), or systemic administration of 1-methyl-4-phenyl-1,2,3,6-tetrahydropyridine (MPTP) in mice and non-human primates (Dluzen and Horstink, 2003; Miller et al., 1998), as well as centrally in non-human primates (Fox and Brotchie, 2010; Morissette and Di Paolo, 2009). Both toxins selectively kill DAergic cells through a combination of excessive generation of oxidative stress and inhibition of mitochondrial respiration (Blum et al., 2001; Glinka et al., 1996; Simola et al., 2007). Methamphetamine (MA) has also been used to create nigral lesions in mice, although the similarities to parkinsonian degeneration have been less well characterised (Dluzen et al., 2002; Dluzen and Liu, 2008).

### 3.2 Sex differences

In studies using genetic animal models of PD, sex differences have thus far received little attention. However, it was reported that the higher expression of certain anti-apoptotic and anti-oxidant molecules found in the striatum of female, wild-type mice compared with males, was lost in *parkin*-null mice, as was the ability of estradiol to stimulate neuroprotective mechanisms in fetal DAergic neurons (Rodriguez-Navarro et al., 2008). Such observations provide clues as to the genetic-molecular processes which appear to differ in males and females, and serve to caution against drawing general conclusions from genetic models where only one sex has been investigated.

**Fig. 1:**
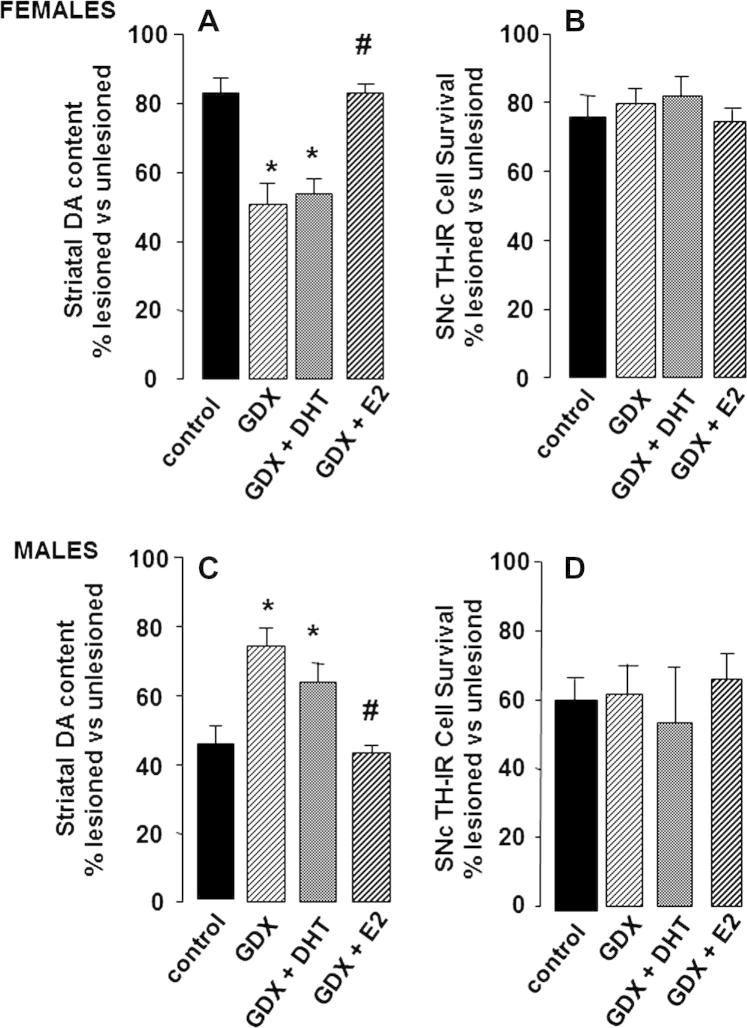
Hormonal influences on 6-OHDA-induced lesions in the nigrostriatal dopaminergic pathway. Male and female rats underwent bilateral gonadectomy (GDX) or sham operation (controls). While still under anaesthesia, animals received a subcutaneous implant of slow release pellets containing estradiol (GDX + E2 to replicate proestrous levels), 5α-dihydrotestosterone (GDX + DHT to replicate physiological androgen levels) or a placebo pellet (GDX group). One week later all animals received a unilateral injection of 1 μg 6-OHDA into the left medial forebrain bundle and 2 weeks later tissue was collected for measurement of the lesion size in the striatum (dopamine, DA, levels in the lesioned side expressed as a percentage of levels in the contralateral, unlesioned side) or substantia nigra pars compacta, SNc (the number of tyrosine hydroxylase immunoreactive, TH-IR, cells in the the lesioned side expressed as a percentage of levels in the contralateral, unlesioned side), as described in Table 1. *Females:* (A) GDX enhanced striatal DA loss and this effect was reversed by E2, not DHT, whereas (B), the loss of TH-IR cells in the SNc was unaffected by hormonal manipulations. *Males:* (C) GDX reduced striatal DA loss and this effect was reversed by E2, not DHT, whereas (D), the loss of TH-IR cells in the SNc was unaffected by hormonal manipulations. Values represent the mean ± s.e.m. (*n* = 6). ^*^ *P* < 0.05 versus gonad intact controls; ^#^ *p* < 0.05 versus GDX. Full details in Murray et al. (2003) and McArthur et al. (2007).

Using toxin-induced models of PD, evidence from our own and other laboratories shows that they are able to reproduce sex differences in disease susceptibility seen in humans. For example, 2 weeks after administration of 1 μg 6-OHDA into the medial forebrain bundle, the depletion of both DA levels in the striatum and loss of DAergic neurons in the SNc is significantly greater in male rats compared with females (Table 1 and Fig. 1 control groups) (Murray et al., 2003; Gillies et al., 2004; McArthur et al., 2007). The progressive loss of DAergic cells over a 5 week period post-lesioning was also consistently greater in 6-OHDA-treated male rats compared with females, confirming a true sex difference rather than a difference in the rate of neurodegeneration (Moroz et al., 2003). Greater SNc and striatal lesions are also seen in male mice compared with females after treatment with MPTP or MA (Liu and Dluzen, 2007; Miller et al., 1998; Dluzen, 2000; Yu and Liao, 2000). Importantly, these sex differences are present only following partial lesions (<60%) of the NSDA pathway using moderate concentrations of 6-OHDA (Gillies et al., 2004; Gillies and McArthur, 2010a); however, when the lesion exceeds approximately 70–80%, sex differences are no longer apparent (Gillies et al., 2004; Gillies and McArthur, 2010a) (Table 1). Collectively, these pre-clinical findings corroborate the clinical studies which suggest that females may be more able to resist the onset and/or progression of neurodegenerative lesions. Moreover, they also demonstrate that the degree of intrinsic neuroprotection in the female brain can be over-ridden once neurodegeneration reaches a certain extent. Additionally, these studies demonstrate that the use of an experimental regimen producing sub-maximal lesions in the NSDA pathway, which is considered to be a model of pre-clinical/early stage PD (Schwarting and Huston, 1996), holds potential for investigating the factors that provide the female advantage.

## 4 Systemic sex steroid hormones and susceptibility in experimental Parkinson’s disease

As might be predicted from clinical observations, most attention has focused on sex hormones as being the major factors driving sex differences in PD. Although males exhibit greater susceptibility, the majority of studies centre on the neuroprotective effects of estrogens in females. However, the available evidence indicates that gonadal factors also play a significant, albeit different, role in males (Gillies et al., 2004; McArthur et al., 2007; Gillies and McArthur, 2010b), meriting separate consideration of the sexes.

### 4.1 Striatal lesions in females

Compared with gonad-intact female rats, the loss of striatal DA induced by 6-OHDA is far greater in ovariectomised rats, and this effect of gonadectomy can be reversed by replacement of estradiol to physiological levels, prior to lesioning (Dluzen, 1997; Murray et al., 2003; Ferraz et al., 2008) (Fig. 1A). Similar preservation of striatal DA by estradiol in females can be demonstrated for NSDA lesions produced by MPTP in mice (Dluzen and Horstink, 2003; Miller et al., 1998) and in non-human primates (Morissette and Di Paolo, 2009), as well as by MA in mice (Yu and Liao, 2000). Smaller losses of striatal DA are also seen when any of these three toxins are administered at the stage of the estrous cycle when estradiol levels are maximal (proestrus), compared with administration when circulating estradiol reaches a nadir (diestrus) (Dluzen and Horstink, 2003; Yu and Liao, 2000; Datla et al., 2003). However, when lesion size exceeds ∼60%, estradiol loses its protective capacity (Table 1) (Murray et al., 2003; Gillies et al., 2004), reminiscent of the loss of advantage in the gonad-intact female with larger lesions (Cordellini et al., 2011). It would appear that this effect may not be off-set by increasing the dose of estradiol, because replacement in ovariectomised rats with levels exceeding the normal physiological range failed to protect, and even exaggerated lesion size (Bourque et al., 2009; Cordellini et al., 2011; Ramirez et al., 2003). To date, the majority of studies discussed here replace estrogens either at the time of ovariectomy or soon after, with evidence to suggest that estrogen administered after creating the NSDA lesion, failed to protect (Bourque et al., 2009; Datla et al., 2003). Collectively, these studies suggest that the level of circulating estradiol prevailing at the time that the lesion is made is critical, and that physiological levels, whether endogenous or achieved through exogenous administration, have protective capacity in the partially injured female NSDA system. Therefore, this supports the view that sex differences in PD could be attributable largely to the protective effect of the gonadal factor, estradiol.

### 4.2 Striatal lesions in males

In contrast to females, striatal DA loss induced by sub-maximal doses of 6-OHDA was significantly reduced in gonadectomised male rats compared with gonad-intactmales (Fig. 1C) (Murray et al., 2003). Although some studies in mice have not detected a significant effect of castration in neurotoxin-induced striatal DAergic lesions (Dluzen et al., 1994; Yu and Wagner, 1994; Gao and Dluzen, 2001), testosterone treatment of gonad-intact or gonadectomised CD-1 male mice, worsened striatal DA loss in animals with mild/moderate lesions (∼40%), but caused no further worsening once the lesions were more extensive (∼70%) (Dluzen et al., 1994; Lewis and Dluzen, 2008). On balance, these findings suggest that, across species, testosterone has a detrimental effect in experimental PD. However, similar to the protective effect of estradiol in females, hormonal modulation is only seen under experimental conditions that mimick the pre-clinical/early phases of the degenerative process. In our own studies, which investigated hormone replacement in 6-OHDA-lesioned castrated rats, we found that administration of the non-aromatisable androgen, dihydrotestosterone (DHT), failed to reverse the effects of gondectomy on striatal DA loss, whereas estradiol treatment did (Fig. 1C). This result suggests that circulating testosterone promotes NSDA degeneration in male rats only after it is converted to estradiol by aromatase enzymes, which are expressed in many brain regions, including the striatum (Kuppers et al., 2000). Data from male mice are more equivocal, with reports that estradiol lacks protective effects against MA toxicity (Gao and Dluzen, 2001; Yu et al., 2002), whereas it may have protective effects against MPTP in males of the highly susceptible C57Bl/6 mouse strain (Dluzen, 1996). Differences in strain, neurotoxin and hormonal treatment regimens are therefore likely to contribute to the variability in the data obtained regarding hormonal influences in male susceptibility for developing PD.

The preceding evidence from experimental models of PD supports the consensus view from clinical observations that ovarian factors are protective in the female NSDA, whilst revealing that gonadal factors may be detrimental in males. This suggests that hormonal influences in both sexes compound to create differences in susceptibility to striatal lesions. Furthermore, they identify estradiol as having dimorphic effects on striatal DA loss in experimental PD in females (reduced striatal lesions) and males (increased striatal lesions), highlighting that the reported clinical benefits of estrogens in the female sex may not simply translate to the opposite sex.

## 5 Hormonal mechanisms underpinning sex differences in experimental PD

### 5.1 The DAT hypothesis

**Fig. 2:**
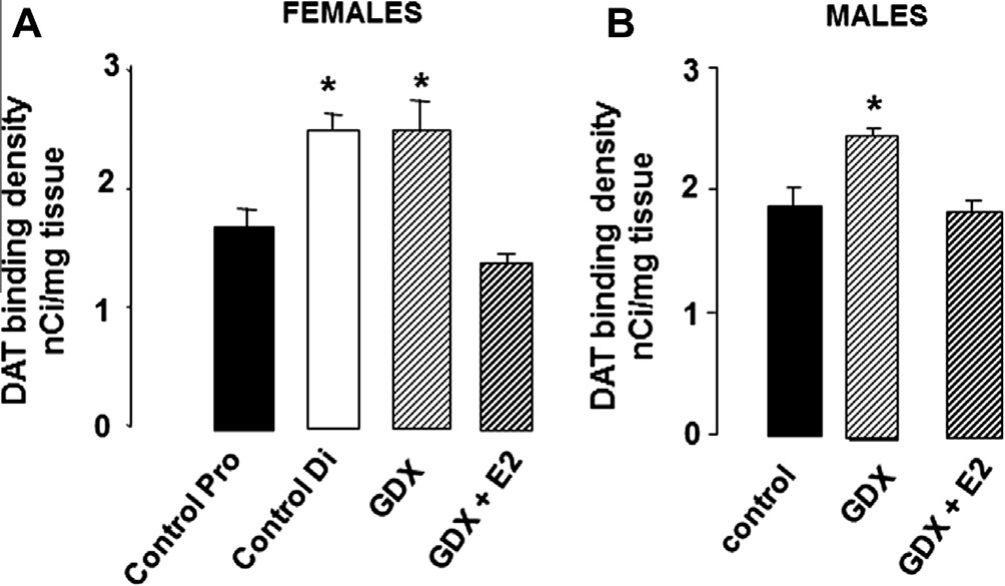
Effects of hormonal environment on striatal dopamine transporter (DAT) levels in (A) female and (B) male rats. Bilateral gonadectomy (GDX) and hormone treatments were performed as described in Fig. 1. Specific binding density of the DAT ligand, [^125^I]-RTI 121, was assessed by autoradiography 1 week later. DAT binding density was similar in control females at proestrus (Control Pro, high endogenous estradiol) and control males, and these values were significantly lower than those found in diestrus females (Control Di, low endogenous estradiol) and after GDX (males and females). Estradiol treatment (GDX + E2) suppressed DAT binding density to proestrus levels in females and to control levels in males. Values represent the mean ± s.e.m. (*n* = 6). ^*^ *P* < 0.05 versus Control Pro (females), controls (males) or GDX + E2 (males and females). Full details in McArthur et al. (2007).

A number of studies suggest that the DA transporter (DAT) acts as a vulnerability factor in PD. DAT-dependent re-uptake of DA from the synaptic cleft is followed by intraneuronal DA metabolism; this leads to the generation of oxidative free radicals, which have potentially damaging effects, and dysregulation of this process may contribute to neurodegeneration in PD. Neurotoxins that are implicated as risk factors in clinical PD (Das et al., 2011; Marsden and Jenner, 1987), or are used in experimental PD (Section 3.1), also have to enter DA neurons via DAT before exerting their damaging effects on mitochondrial respiration, with concomitant excessive generation of oxidative stress (Gainetdinov et al., 1997; Miller et al., 1999). Therefore, it has been proposed that the protective effects of estradiol may be mediated by suppressive effects on DAT (Dluzen, 2000). In accord with this hypothesis, we have shown that in gonad-intact females striatal DAT levels are significantly higher at diestrous (low endogenous estradiol levels) compared with proestrous (high endogenous estradiol levels) (Fig. 2A) (McArthur et al., 2007), and this coincides with a greater depletion of striatal DA when the neurotoxin is administered at proestrus compared with diestrus (Datla et al., 2003) {McArthur, 2007 #715} (Yu and Liao, 2000). Furthermore, ovariectomy increased both striatal DAT binding, by ∼50% (Fig. 2A), as well as DA depletion (Fig. 1A), with both effects being reversed by estradiol replacement (Figs. 1A and 2A) (Murray et al., 2003; McArthur et al., 2007). However, the DAT hypothesis does not hold true in males, and fails to provide an explanation for sex differences in PD. For example, striatal DAT levels in male rats were the same as those in females at proestrus (Fig. 2), although 6-OHDA-induced striatal DA depletion was significantly greater when the toxin was administered to gonad-intact males compared with female at proestrus (Fig. 1A). Moreover, contrary to our prediction that castration in males would induce a decrease in striatal DAT levels as an explanation for the reduction in 6-OHDA-induced striatal lesions seen in gonadectomised males (Fig. 1C), DAT binding density actually increased (by ∼20%) (Fig. 2B) {McArthur, 2007 #715}. It would follow from these observations that the response of the NSDA to injury is sexually dimorphic.

### 5.2 Compensation versus neuronal survival

Depletion of striatal DA in PD is presumed to reflect degeneration and loss of DAergic cells in the SNc, so one would expect that the marked effects of gonadectomy and hormone treatments on striatal DA lesions in experimental PD would be reflected by qualitatively similar changes in DA perikarya in the SNc. However, despite the substantial evidence that systemic hormonal status affects striatal DA loss (Sections 4.1 and 4.2; Fig. 1A and C), the survival of TH-IR cells in the partially lesioned SNc was unaffected in either sex, whether animals were gonad-intact, gonadectomised or gonadectomised and treated with estradiol, DHT or vehicle (Fig. 1B and D) (McArthur et al., 2007; Moroz et al., 2003; Ferraz et al., 2003, 2008). Additionally, estradiol failed to protect against 6-OHDA-induced DA cell loss in primary, serum-free mesencephalic cultures (Callier et al., 2002). These findings therefore suggest the unexpected conclusion that the pronounced, yet qualitatively different effects of the sex hormone environment in males and females on toxin-induced striatal DA depletion must occur independently of DAergic cell survival in the SNc.

**Fig. 3:**
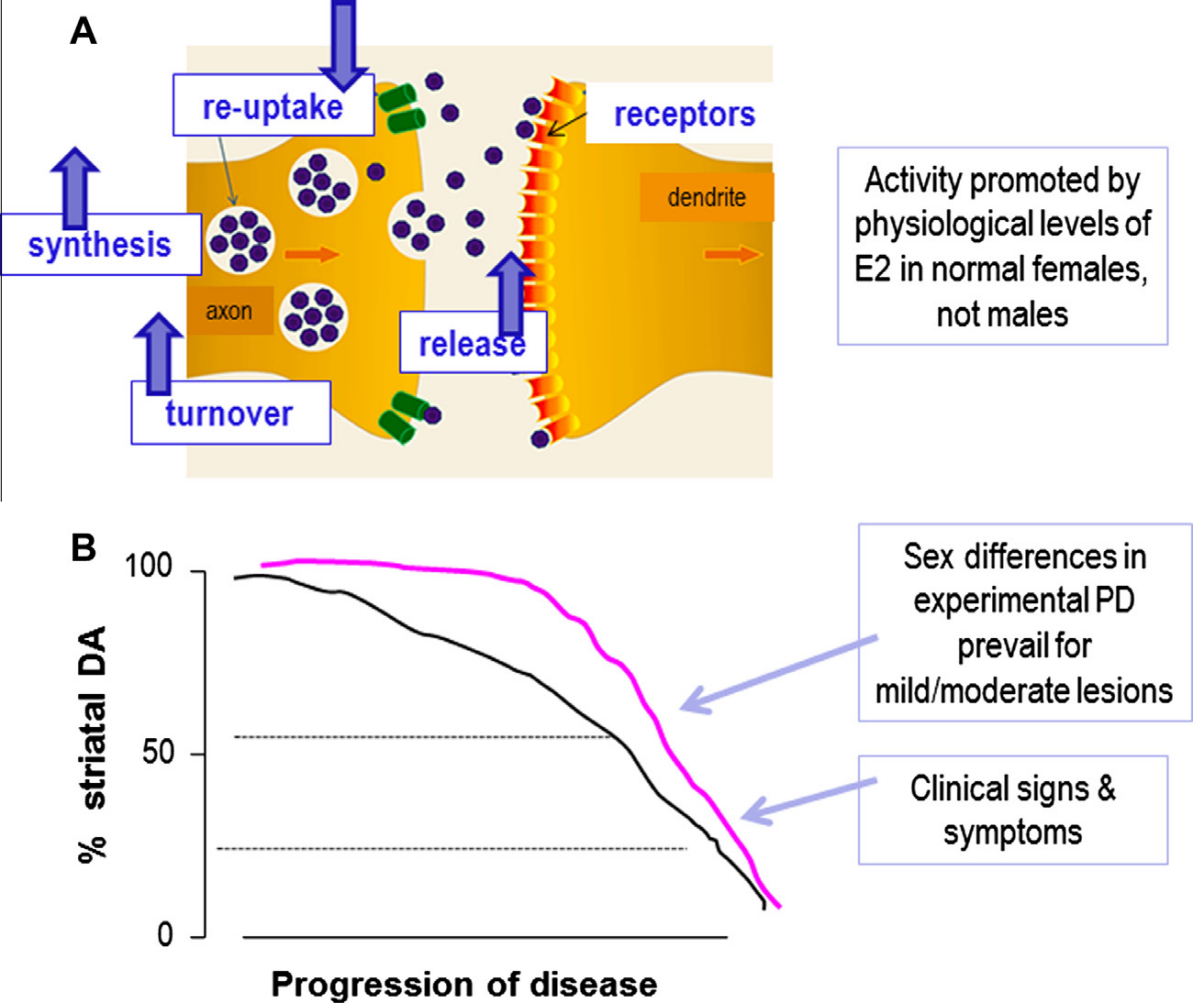
Hypothetical schema for estrogenic influences on compensatory mechanisms in striatal terminals of surviving neurons in the damaged nigrostriatal dopaminergic system. (A) As indicated by the arrows, in females, but not in males, estradiol (E2) enhances dopamine synthesis, release and turnover, whilst suppressing DAT levels and re-uptake of the neurotransmitter from the synaptic cleft. (B) This preserves striatal function when lesion size is small/moderate (⩽50%), but fails to do so as lesions increase, allowing motor symptoms to become manifest. This action of E2 in females may render them more able to delay progression, or even onset, of disease.

The foregoing discussion raises two important questions: how can the effects of physiological levels of circulating estradiol at the nerve terminal of the partially injured NSDA system be dissociated from effects at the level of the cell body in both sexes, and why should the effects at striatal level be different in males and females? We have proposed that sexually dimorphic hormonal influences on the activity of the neurons which survive in pre-clinical/earlyPD, rather than on neuron survival *per se*, provides a compelling explanation for these observations (McArthur et al., 2007; Gillies and McArthur, 2010a,b). This concept is summarised in Fig. 3, and supported by the following evidence.

In both clinical and experimental PD, the brain has a remarkable capacity for compensation, and this is thought to account for the fact that overt motor symptoms may not be apparent until around 80% of striatal DA and 60% of SNc DAergic perikarya are lost (Bassilana et al., 2005; Bezard et al., 2001, 2003; Castaneda et al., 1990; Song and Haber, 2000). The compensatory mechanisms are complex and not fully understood, but are likely to involve adaptations within the surviving DAergic neurons, such as increased synthesis, metabolism and release of DA, to compensate for cell loss. Importantly, evidence from biochemical and behavioural studies indicates that females have an improved capacity to recover from toxin-induced PD compared with males (Tamas et al., 2005; Bourque et al., 2012), suggesting a sex difference in the compensatory mechanisms. Furthermore, the sensitivity of these compensatory mechanisms to sex hormone influences may be sexually dimorphic. For example, in female, but not male rodents, circulating estradiol (endogenous and exogenously given) promotes basal, potassium- and amphetamine-stimulated striatal DA release *in vivo* and *in vitro* (Becker, 1999; Dluzen, 2005; McDermott et al., 1994; Ohtani et al., 2001; Pasqualini et al., 1995; Serova et al., 2004; Xiao and Becker, 1998), and promotes *in vivo* stereotypic behaviours involving the NSDA system (Becker, 1999). Estradiol also increases DA turnover in females, but not in males, and is more effective in the female compared with the male striatum in suppressing the density of DAT (Fig. 2) (McArthur et al., 2007), which critically regulates DAergic neuron dynamics. Estrogens could be exerting these effects via direct actions on the DAergic neurons themselves, but indirect effects on input circuitry are also feasible. Of particular interest is the locus coeruleus, which is known to influence the NSDA system and its adaptive response to injury via noradrenergic transmission (Marien et al., 2004). Notably, circulating estradiol has been reported to up-regulate expression of TH (the rate-limiting enzyme for nor-adrenaline as well as DA synthesis) in the female locus coeruleus, whereas in males circulating testosterone down-regulates TH only after its conversion to estradiol (Thanky et al., 2002). These sexually dimorphic hormonal responses have striking parallels with the effects of hormonal manipulations on striatal DA depletion in experimental PD (Section 4). Therefore, we propose that circulating gonadal steroids, acting indirectly at the locus coeruleus to modify nor-adrenergic regulation of the NSDA system in a sex-specific manner, offers a potential mechanisms to explain why the effects of estradiol on 6-OHDA-induced striatal DA loss is different in males and females (Gillies and McArthur, 2010a). Other sexually differentiated, estrogen-sensitive networks which could modify adaptations of the NSDA system to injury include the mesocortical DAergic pathway (Kritzer and Creutz, 2008) and the serotonergic system of the dorsal raphe (Klink et al., 2002). Further studies are needed to establish the role of these systems in clinical and experimental PD.

Collectively, these findings demonstrate that in females, estrogens have a positive effect on mechanisms underpinning compensatory processes in the injured NSDA, which could thus explain why estradiol appears neuroprotective at the level of DAergic terminals, but not the perikarya. The failure of estradiol to have such effects in male rodents is compatible with its failure to protect against striatal lesions in this sex (Murray et al., 2003; Gao and Dluzen, 2001; Disshon and Dluzen, 2000), and suggests mechanisms which could underpin sex differences in PD.

### 5.3 Innate sex differences in NSDA and related circuitry

Similar to the human NSDA system (discussed in Section 2.2), biological sex differences are also present in the rodent NSDA system. This is supported by studies showing sex differences in hormonal responsiveness of NSDA circuitry, described above (Section 5.2). Additionally, the male SNc contains significantly more DA neurons with a different topographical organisation compared to the female SNc (Dewing et al., 2006; Murray et al., 2003; McArthur et al., 2007). Yet, despite these structural differences, no sex differences are reported for striatal DA content and basal extracellular striatal DA levels, which are thought to be maintained by sex differences in neuronal dynamics (Murray et al., 2003; Robinson et al., 1990; Walker et al., 2000; Ji and Dluzen, 2008; Virdee et al., 2013). However, amphetamine-stimulated DA release and DA-dependent motor responses are significantly greater in females compared with males (Becker, 1999; Virdee et al., 2013), indicating functional differences when the system is activated. Interestingly, equalisation of male and female circulating sex hormones by gonadectomy, generated marked sex differences in extracellular striatal DA levels, which were absent in the gonad-intact animals (Walker et al., 2000). This suggests that there are fundamental sex differences in the organisation of the NSDA-associated circuitry, which are independent of the adult sex hormone environment.

The origins of sex dimorphisms in the NSDA system have received relatively little attention, but the organisational actions of gonadal hormones during development, as well as genetic factors (see Section 6.1), are likely to have important influences (Dewing et al., 2006; Gillies et al., 2004; Gillies and McArthur, 2010b; Vadasz et al., 1985, 1988). Regarding hormonal factors, it is well established that a transient perinatal rise in testosterone production by the developing testes is a principal factor in the masculinisation/defeminisation of rodent brain regions controlling reproduction and reproductive behaviours (McCarthy, 2008; Wilson and Davies, 2007; Arnold and Breedlove, 1985). Most evidence for this concept relates to the hypothalamus, which has been the prototype for studying brain sex differences. Significantly, the sexually differentiating effect of testosterone in the brain occurs largely after its conversion to estradiol by brain aromatases. This early exposure to estradiol is thought to result in a loss of capacity of the male brain to respond to estradiol in later life, whereas the female brain retains this responsiveness to estradiol, as manifest by the ability of estradiol to stimulate ovulation by positive feedback effects within the hypothalamo–pituitary–gonadal axis. Hence, defeminisation of the brain has also been defined as a loss of capacity to respond to oestrogens (McCarthy, 2008). Interestingly, one study reported that neonatal treatment of female rats with testosterone, to mimic the transient elevations occurring naturally in males, compromised the protective effect of estradiol in the 6-OHDA model of PD (Moroz et al., 2003). These observations support the notion that the adult male NDSA system, whether intact or injured, may also lose its capacity to respond to estradiol as a result of early exposure to estradiol after aromatisation of testosterone. This intriguing mechanism, which could explain the sex dimorphism in estrogen’s ability to protect against striatal DA depletion in experimental PD, remains to be fully tested.

### 5.4 Estrogenic signalling

Thus far, we have considered ways in which physiological levels of sex hormones, particularly estradiol, differentially influence adaptive/compensatory mechanisms in the partially injured NSDA in males and females, thereby affecting striatal DA levels, but not DAergic cell numbers in the SNc (Sections 2.3.3 and 4). Such mechanisms of estrogenic protection relate specifically to situations where NSDA lesion size is mild/moderate and will be unique to PD, rather than being more widely applicable to other forms of brain injury. However, estradiol also exerts more generally acclaimed neuroprotective actions which can prevent cell loss in many types of brain injury. These include ischaemia, trauma, infection and neurodegeneration, where estrogens can activate various non-specific processes involved in cell survival, such as mitochondrial function, anti-oxidant activity and effects on gene products involved in regulating apoptosis. These mechanisms, along with considerations of whether estradiol’s protective effects involve the classical nuclear estrogen receptors (ER), ERα or ERβ, or the more recently recognised G protein-coupled receptor, GPER1 (also termed GPR30), are covered in many extensive reviews (Bourque et al., 2012; McEwen and Alves, 1999; Brann et al., 2007; Wise et al., 2001; Garcia-Segura et al., 2001; Raz et al., 2008; Behl, 2002; Green and Simpkins, 2000). Therefore, here we shall comment only on the relatively sparse information which may be relevant to PD and sex differences.

Whether or not estradiol protects against SNc DAergic cell loss in experimental PD appears to vary with the neurotoxin used, the severity of the lesion, the strain and species of rodent and the treatment regimen (dose of estrogen used; period of hypoestrogenicity between ovariectomy and treatment), as well as sex (Gillies and McArthur, 2010a; Ferraz et al., 2008; Cordellini et al., 2011; Brinton, 2008; McArthur and Gillies, 2011). For example, in our protocol, where estradiol failed to prevent SNc DA cell loss, the steroid was replaced at the time of ovariectomy using slow release implants to provide proestrus levels (circa 200 pmol estradiol/ml); unilateral injections of 1 μg 6-OHDA were given 1 week later to produce a mild/moderate lesion, followed by a 2 week interval for the lesion to develop before tissue was collected for analysis (Murray et al., 2003; McArthur et al., 2007) (Fig. 1, Section 4.1). In contrast, another study reported significant protection at both SNc and striatal levels when using commercially ovariectomised rats; after an undefined period of hypogenicity, a bolus injection of estradiol (20 μg s.c.) was administered 24 h prior to unilateral injections of 8 μg 6-OHD to produce a very large lesion (>90% loss of striatal DA and >60% loss of SNc DAergic neurons) (Quesada et al., 2008). These comparisons highlight the complexity of estrogenic protective effects and our present inability to reach a simple consensus regarding the issue of hormonal protection in PD. However, the work by Quesada and colleagues (Quesada et al., 2008), using female rats, demonstrated that DA cell survival was dependent on estradiol acting via the IGF-1 system operating through the phosphatidylinositol 3-kinase (PI3K)/Akt (PKB) pathway, which modulates expression of anti-apoptoic (Bcl-2) and apoptotic (Bad, Bax) proteins to promote cell survival. This is in agreement with reports of the importance of IGF-1 as a mediator of estrogenic neuroprotection in many circumstances (Mendez et al., 2005). However, it remains to be determined whether sex differences exist in estrogenic neuroprotection mediated via IGF-1 and its down-stream signalling. Indeed, this should not be ruled out, because evidence suggests that intracellular signalling pathways may be sexually differentiated as a result of perinatal exposure to raised endogenous estradiol levels, after aromatisation of testosterone, in males, at least in the hypothalamus (Abraham and Herbison, 2005; Auger et al., 2001).

Interesting sex differences have, however, been noted in the expression of molecules associated with cell survival pathways, both in the intact and injured SNc. For example, compared with males, female C57Bl/6 mice possess greater Bcl-2/Bax and glutathione/glutathione disulphide ratios in the striatum, suggesting a balance of pro-survival and anti-oxidant factors, respectively, to favour an inherent superior neuroprotective capacity (Rodriguez-Navarro et al., 2008). Sex differences have also been reported in the striatum for the temporal expression of molecular pathways involved in cell survival as the DA response to the neurotoxin, MA, develops over 3 days (Bourque et al., 2011). Hence, an initial fall in striatal levels of DA and phosphorylated Akt in both sexes (30 min after MA injection) was associated in females, but not in males, with increases in levels of extracellular signal-regulated kinase (ERK1/2) (30 min), Akt (day 1) and phosphorylation on serine 9 of glycogen synthase kinase 3β (GSK3β; day 1 and 3); by day 3 DA levels had risen significantly in females, whereas they remained significantly lower in males. Activation of GSK3β, a constitutively active kinase, is associated with neuronal apoptosis caused by oxidative stress (Gomez-Sintes et al., 2011), which is a key neuropathological process in clinical and experimental PD (Phani et al., 2012). Both the PI3K/Akt and the ERK1/2 pathways converge downstream on GSK3β to inhibit its activity by phosphorylation on serine 9 (Manning and Cantley, 2007). Together, the sex-specific striatal molecular patterns following MA administration suggest a reduction in GSK3β activity in females, favouring recovery in females, but not in males. However, the precise striatal cell types involved in these changes remains to be determined, as does the relevance of the striatal data for nigral cell loss. An effect at the level of the DA neuron is supported by the finding that GSK3β activation (via Ser 9 dephosphorylation) mediated 6-OHDA-induced death in susceptible cell lines (Chen et al., 2004). Additionally, a recent study using male rats reported that intrastriatal injection of 6-OHDA led to activation of GSK3β and caspase 3 in SNc DA neurons, suggesting activation of the intrinsic (mitochondrial) pathway of apoptosis (Hernandez-Baltazar et al., 2013). Future studies investigating the influences of sex steroids on these pathways in females as well as males will provide important insight into sex differences in PD and the potential for sex-specific therapies.

In the monogenetic forms of PD, mutations frequently occur in genes encoding proteins that localise to the mitochondria, including PINK-1, DJ-1, α-synuclein and LRRK2 (Canet-Aviles et al., 2004; Devi et al., 2008; Gautier et al., 2008; Papkovskaia et al., 2012). Deregulation of mitochondrial function has also been linked to sporadic forms of PD, either as a potential direct cause or indirect consequence of neuronal damage. The contribution of mitochondrial dysfunction to DAergic degeneration is corroborated in toxin-induced animal models of PD (Dexter and Jenner, 2013), supporting the view that mitochondrial regulation of cell survival molecules (including those discussed above) and apoptosis is compromised in PD. Interestingly, in non-neuronal cell lines ER ligands can influence the intrinsic (mitochondrial) apoptotic pathway, as well as the extrinsic apoptotic pathway, and this depends on their relative affinities for ERα and ERβ and the ratio of expression of ERα:ERβ within the cells (Pons et al., 2013; Chen and Chien, 2013). Moreover, mitochondrial ERα and ERβ have been shown to act in co-operation with the nuclear ERs to regulate the mitochondrial respiratory chain (Chen et al., 2009). Thus, although sex differences in mitochondrial function have not yet been linked to a sex bias in PD, the observation that apoptotic mechanisms are sensitive to prevailing levels of sex hormones suggests that such a possibility merits further investigation.

Sex differences in the levels of ERs within the NSDA pathway could potentially underlie sex differences in estrogenic signalling pathways. Separate studies using the same antibody reported that ERβ was absent in the male mouse SNc (Shughrue, 2004), but weakly expressed in the female SNc (Mitra et al., 2003; Merchenthaler et al., 2004); the striatum appears not to express ERβ (Shughrue, 2004). ERα was not found in the DAergic neurons of the SNc and its expression in the striatum is low, although possibly at a higher level in female compared with male mice (Rodriguez-Navarro et al., 2008). Current evidence therefore provides minimal support for sex differences in ER expression, and supports the view that estrogens may act indirectly or via nuclear receptor-independent mechanisms to influence DAergic neurons. Supporting this view, single sex studies using ER selective ligands or ER null and wild type mice found that ERα, ERβ and GPER1 play a role in estrogenic neuroprotection, with ERα possibly playing the more dominant role (Al-Sweidi et al., 2011; Bourque et al., 2013; Baraka et al., 2011). Further studies are needed to determine whether selective ligands may exert sex-specific effects.

## 6 Genes, environment and sex differences in experimental Parkinson’s disease

### 6.1 The *SRY* gene

The results discussed above (Section 5, Table 1, Fig. 1) demonstrate that, although there is a clear sex difference in the survival of SNc DAergic neurons in experimental models of the pre-clinical/early stages of PD, this occurs independently of sex hormone influences (McArthur et al., 2007; Moroz et al., 2003). The origins of these differences remain unknown. However, in the intact adult SNc, we and others have reported that the numbers of DAergic neurons are significantly greater in male rats and mice compared with female rodents by ∼20% (Dewing et al., 2006; Murray et al., 2003; McArthur et al., 2007). This is perhaps surprising, considering the fact that a greater percentage of cells are lost in the male SNc compared to females. A possible explanation could be the existence of a heterogeneous population of cells within the SNc, a notion supported by the observation that there are regional differences in resistance or resilience of DAergic cells in rodent models of PD, as well as in PD patients (Gillies et al., 2004; McArthur et al., 2007; Damier et al., 1999; Carman et al., 1991). A greater proportion of resilient cells in females and/or a lower proportion of this resilient population in males are thus plausible explanations for sex differences in PD.

Our own studies rule out the possibility that sex hormones play a significant role in determining the size of the adult DAergic population in the SNc. Hence, we have shown that neither adult gonadectomy nor neonatal gonadectomy of newborn male rats, with or without the masculinising influence of neonatal treatment with DHT or estradiol, affects the adult DAergic neuron count (Gillies et al., 2004; McArthur et al., 2007). Nigral cell number does, however, appear to depend on expression of SRY coded by the Y chromosome, which has been co-localised in a subset of male SNc DA neurons (Dewing et al., 2006). Silencing of the *SRY* gene in the male rodent SNc reduces the DA neuron number to that of females and induces motor deficits. These findings therefore support the emerging concept that factors encoded by the sex chromosome genes, as well as sex hormones, contribute to sexual differentiation of the brain (Arnold and Burgoyne, 2004; Arnold, 2012). It remains to be determined whether SRY influences susceptibility of DA neurons to injury, but it does represent an interesting non-hormonal candidate responsible for sex differences affecting DAergic cell loss in the SNc, at least in experimental PD.

### 6.2 Stress and early life adversity

The impact of adverse environments in experimental PD is an understudied area. Our preliminary evidence suggests that exposure of adult rats to immobilisation stress exacerbates striatal and nigral lesions produced by 6-OHDA in males, but not in females (unpublished observations, Allen, Buckingham, Gillies & Dexter). In addition, studies performed in male animals suggest that exposure to environmental stressors in early life puts the midbrain DAergic neuronal population at risk in later life.

For example, perinatal exposure to inflammatory stimuli (lipopolysaccharide, the bacterial endotoxin), obstetric complications, pesticides and iron-enriched diets, may themselves alter adult midbrain DAergic neuron number and activity, and/or enhance their susceptibility to subsequent challenges, including exposure to 6-OHDA (Wang et al., 2009; Barlow et al., 2007; Ling et al., 2004; Boksa, 2004; Boksa and El-Khodor, 2003). In addition, neonatal separation of male rat pups from their mothers exacerbated the toxic effects of 6-OHDA on locomotor activity and striatal TH expression in adulthood (Pienaar et al., 2008). As responses both to acute and chronic stressors in adulthood and to early-life adversity are sex-specific (Kudielka and Kirschbaum, 2005), the impact of stress throughout life for developing PD, as well as being a potential source of sex differences in PD susceptibility, requires attention.

## 7 A role for glia

Astrocytes and microglia are the major glial cell types in the brain and comprise 80–90% of the total brain cellular population (Schwarz and Bilbo, 2012). They play critical, co-operative roles in maintaining CNS neurotransmission and homeostasis, as well as mounting innate immune responses aimed at protecting the brain against insults such as physical trauma, ischaemia, infection and neurodegenration (Klegeris et al., 2007; Halassa and Haydon, 2010). In the early, activation stage of the innate immune response, microglia and to a lesser extent, the astrocytes, produce oxidative and pro-inflammatory (potentially harmful) mediators; this triggers the later, resolution phase of the immune response, involving production of anti-inflammatory (protective) factors as well as the induction of phagocytic microglia, a phenotype essential for clearance of dying or dead cells and termination of the inflammatory response (Farina et al., 2007; Minghetti, 2005; Lucas et al., 2006). However, excessive or chronic glial activation and/or failure to resolve the neuroinflammatory response can become damaging and can exacerbate on-going disease processes, including those relating to PD (Rogers et al., 2007; McGeer and McGeer, 2008; Barres, 2008). It remains a highly controversial issue as to whether microglial inflammation is a bystander effect of PD-related neurotoxicity or a primary pathogenic process, but animal experimental studies as well as investigations of post-mortem tissues identify microglial activation and neuroinflammation as an early biomarker for disease (Chung et al., 2009; Lundblad et al., 2012). Control of glial activity is thus an attractive target for novel neuroprotective strategies. Furthermore, emerging evidence suggests that the central neuro-immune response is sexually dimorphic (Schwarz and Bilbo, 2012; Santos-Galindo et al., 2011). Glial cells should, therefore, be considered as contributory factors to sex differences in PD.

### 7.1 Astrocytes

Studies of specific hypothalamic nuclei demonstrate that astrocytes are sexually differentiated in adulthood in terms of their morphological complexity, cell signalling mechanisms, plasticity and hormone responsiveness, especially to estradiol (McCarthy, 2008; Naftolin et al., 2007; Kuo et al., 2010). These sex dimorphisms are programmed neonatally by testosterone after conversion to estradiol, and suppress the ability of the adult hypothalamic circuitry to respond to estrogen priming of the gonadotrophin releasing hormone/luteinising hormone surge, which is essential to trigger ovulation. Whether astrocytes are similarly sexually differentiated in other adult brain regions is an important question that needs to be addressed. However, developing astrocytes from many brain regions show sex-specific characteristics. For example, primary cortical astrocyte cultures from newborn male mice and hormonally masculinised newborn females produce a greater cytokine response when challenged with the bacterial endotoxin, lipopolysaccharide, compared with normal female astrocytes, indicating that sex differences in the neuroimmune response may be predetermined by perinatal testosterone exposure (Santos-Galindo et al., 2011). When challenged with MPTP, astrocyte cultures derived from the neonatal male mouse mesencephalon also responded with a greater elevation in oxidative stress levels compared with female mesencephalic astrocytes or cortical astrocytes of either sex (Sundar Boyalla et al., 2011), suggesting that the glial response to neurodegenerative processes is both sex- and brain region-specific, and is most robust in the NSDA system. In adult mice, MPTP administration increased striatal expression of the astrocyte-specific marker, glial fibrillary acidic protein (GFAP) in both sexes, but a time-course study over 21 days revealed a more sustained activation in females (Ciesielska et al., 2009). Although the functional implications require further study, the data suggest that in males astrocytes may generate a greater, potentially harmful, neuro-inflammatory response, whereas in females astrocytes contribute compensatory and survival-promoting properties, favouring more efficient recovery mechanisms, which, together, may contribute to sex differences in PD.

It has long been acknowledged that, across species, there are sex differences in the peripheral immune system. To a large extent, this has been attributed to gonadal hormones, especially estradiol, which augments aspects of immune activity (Schwarz and Bilbo, 2012; Nadkarni and McArthur, 2013). Gonadal hormones also regulate glial response to injury (Arevalo et al., 2013) and, to a large extent, the neuroprotective actions of circulating estradiol are dependent on its powerful anti-inflammatory actions on astrocytes (Garcia-Ovejero et al., 2005; Marchetti et al., 2005; Vegeto et al., 2008). Hence estradiol treatment of midbrain astrocytes *in vitro* reduces the production of cytokines and inflammatory mediators in response to inflammatory stimuli (Vegeto et al., 2008; Kipp et al., 2007), and *in vivo* circulating estradiol down-regulates astrocyte activation in parallel with its protective effects in female mice treated with MPTP (Morale et al., 2006). The effect of androgens on glial activity has received relatively little attention. Available data are contradictory, with evidence for both protective and detrimental effects, depending on the type of injury, among other factors (Arevalo et al., 2013), although the effects on astrocytes in PD models has not been reported. On balance, the available evidence would support the view that astrocytes are important targets for circulating estrogens, which could contribute to sex differences in PD.

In addition to responding to estrogens, astrocytes are capable of synthesising estradiol. In response to injury, aromatase expression is induced *de novo* in astrocytes, enabling relatively high levels of estradiol to be synthesised locally and exert a variety of protective effects on vulnerable neurons (Azcoitia et al., 2010). Although this response occurs in both males and females, recent evidence suggests that it occurs more rapidly and robustly in females (Liu et al., 2007; Mirzatoni et al., 2010), further suggesting a mechanism which could explain the smaller lesion size and faster recovery in females which characterises many types of brain injury, including PD.

Evidence for constitutive expression of ERs in astrocytes is contradictory but, similar to the situation for aromatase, receptor up-regulation, especially for ERα, appears to be a key part of the neural response to many types of brain injury in rodent and primates (Garcia-Ovejero et al., 2005; Blurton-Jones and Tuszynski, 2001). This phenomenon has not been specifically reported for the damaged NSDA system, but, notably, a marked increase in GPER1 was seen in the striatum of male, but not female, mice 7 days after exposure to methamphetamine (Bourque et al., 2011). The specific cell type mounting this response was not identified, and the significance remains to be determined. However, the GPER1 selective agonist, G1, has been reported to protect against MPTP-induced striatal toxicity (Bourque et al., 2012) and, as the G1 ligand lacks estrogenic activity in reproductive tissues, such observations merit further investigation for therapeutic potential (Otto et al., 2009).

Taking the astrocyte response to injury as a whole, it would thus appear that central estrogenic activity could critically regulate progression of the astrocytic activation and resolution through the neuro-inflammatory cascade. Hence, the initial response to a challenge, involving the release of pro-inflammatory cytokines, chemokines and reactive oxygen/nitrogen species, may be primed by circulating estradiol and is necessary for local resistance and repair mechanisms; this is closely associated with an up-regulation of astrocytic estradiol synthesis (aromatase), as well as astrocytic responsiveness to estrogens (ERs)and astrogliogenesis (proliferation and increased GFAP expression), which achieves a local estrogenic environment capable of preventing over-activation of the neuro-immune response and exacerbation of neural damage, whilst also exerting its protective effects on adjacent neurons. The sex differences identified in these mechanisms would clearly contribute to sex differences in PD.

### 7.2 Microglia

**Fig. 4:**
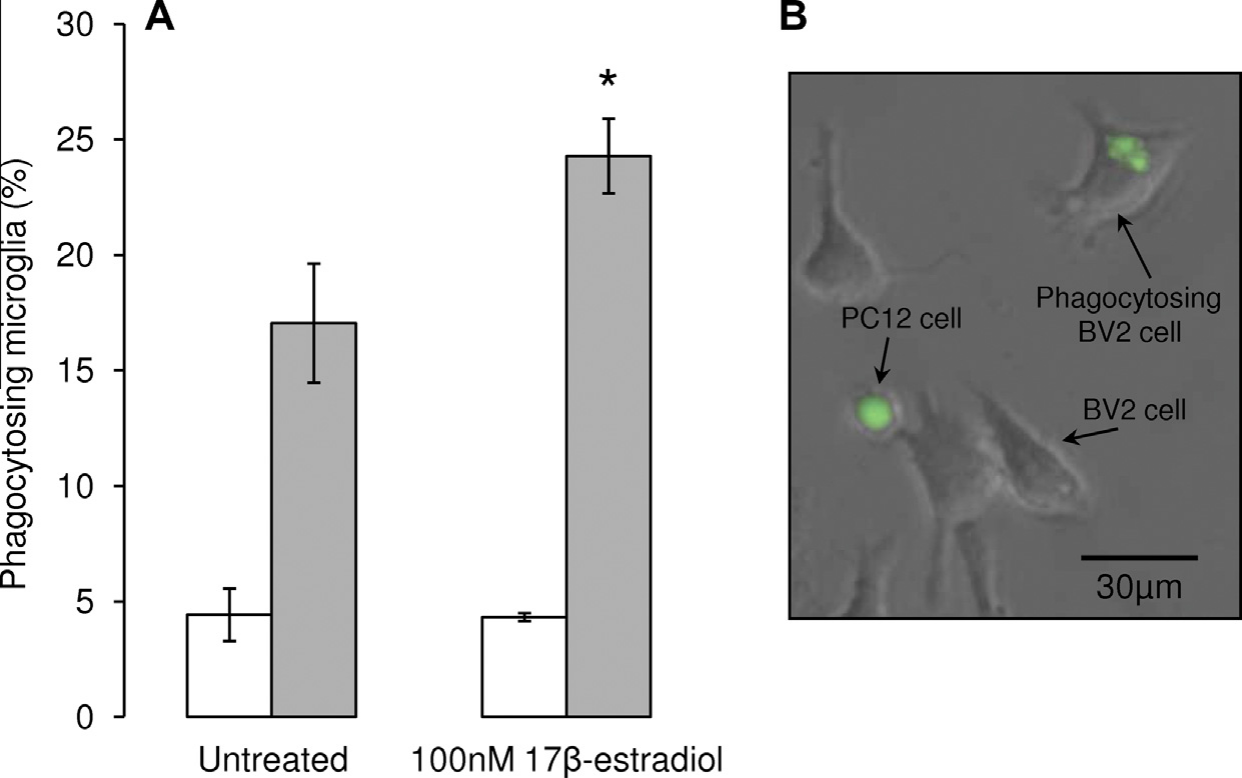
Estradiol enhances microglial phagocytosis. Co-cultures of the murine microglial BV2 cell line and the dopamine-producing neuron-like PC12 cells were used as a model to investigate hormonal influences on microglial phagocytosis of apoptoic neurons (McArthur et al., 2010). (A) Treatment of BV2 microglia for 16 h with 100nM 17β-estradiol had no effect on the phagocytosis of vehicle-treated, non-apoptotic PC12 cells, but significantly increased the number of microglia phagoytosing apoptotic 6-OHDA-treated PC12 cells. ^*^ *P* < 0.05 versus untreated BV2 cells. (B) Example of BV2 microglia phacocytosing CMFDA-labelled green fluorescent PC12 cell treated with 6-OHDA.

Data on sex differences in the numbers or activity of microglia in the adult brain are sparse, but developmental studies suggest that they may be profound, with significance for brain disorders. For example, at postnatal day 4, the numbers of microglia in the male rat hippocampus, parietal cortex and amygdala are significantly greater than those seen in females (Schwarz and Bilbo, 2012). This has been proposed to lead to a fundamentally different neuro-immune response to early-life immune challenge, which has been related to long-term, profound changes in microglial function and cognitive behaviours in males, but not females. Microglia are thought to contribute to DAergic cell damage in clinical and experimental PD (Halliday and Stevens, 2011; McGeer and McGeer, 2008; Orr et al., 2005; L’Episcopo et al., 2010), but whether sexual differentiation of NSDA microglia contributes to sex differences in PD remains to be determined. However, similar to astrocytes, gonadal steroids are powerful regulators of microglial numbers and reactivity in many circumstances (Arevalo et al., 2013). Of relevance to PD, it has been demonstrated that activation of microglia with LPS can cause the death of DAergic neurons in cultures of primary mesencephalic neurons or DAergic cell lines through the release of toxic factors such as reactive oxygen/nitrogen species and TNFα (Liu et al., 2005; McArthur et al., 2010), and this can be prevented by co-treatment of the microglia with estradiol (Liu et al., 2005). Studies *in vivo* also showed that administration of estradiol to ovariectomised mice treated with MPTP suppressed iNOS and nitric oxide production by microglia and protected against striatal DA loss (Morale et al., 2006). In addition to suppressing the potentially harmful phenotype induced by injury, our preliminary data suggest that estradiol can also promote microglial phagocytosis of DAergic PC12 cells rendered apoptotic by 6-OHDA treatment (Fig. 4; McArthur, Vohra, Solito & Gillies, unpublished observations); this occurs via ERβ signalling, whereas GPR30 signalling may suppress microglial phagocytic activity, whilst promoting cell cycle progression and cell proliferation (Vohra et al., 2012), a property of this receptor which has been reported in other tissues (Prossnitz et al., 2008).

## 8 Conclusions

In this review we have summarised the clinical and experimental evidence which demonstrates that males have a greater susceptibility to PD, compared with females. Compelling evidence suggests that biological sex differences in the NSDA pathway may underlie these differences in vulnerability, and could also account for the sexually dimorphic actions of estradiol, which protects females against striatal DA loss in experimental PD, but fails to protect, or may even worsen, striatal lesions in males. These findings open up the potential to exploit hormone-based therapies as a novel approach to develop treatments which can delay, and possibly halt, progression of the disease. Such disease modifying strategies are urgently needed to supersede current treatments relying on DA replacement strategies, which can only ameliorate symptoms. The findings also highlight the need for sex-specific medicines, which demands a better understanding of sex dimorphisms in the intact and injured NSDA. This will also require investigation into how the internal hormonal milieu interacts with other sex-specific factors, including differential exposure to potentially neurotoxic agents, as well as the impact of *SRY* gene expression within SNc DAergic neurons. Although PD research has classically focussed on the NSDA system, other pathways showing signs of pathologic change prior to those in the NSDA are receiving growing attention and may help to explain the non-motor symptoms (Dexter and Jenner, 2013; Jenner et al., 2013; Bronstein et al., 2009). Therefore, as our concept of PD is evolving, future studies on sex differences in the prevalence and nature of PD should spread the net wider.

## Figures and Tables

**Table 1:** Sex differences in the 6-hydroxydopamine (6-OHDA) model of Parkinson’s disease.

MFB injection	Striatal DA content % lesioned versus unlesioned side		TH-IR cells % lesioned versus unlesioned side cells	
	Male	Female	Male	Female
Vehice control	105.4 ± 9.8	100.3 ± 6.2	101.4 ± 8.9	98.6 ± 8.5
1 μg 6-OHDA	50.3 ± 4.8⁎	72.7 ± 6.7⁎^,^#	59.3 ± 4.5⁎	76.2 ± 2.5#
6 μg 6-OHDA	4.2 ± 0.3⁎⁎	5.1 ± 0.3⁎⁎	25.5 ± 8.3	15.7 ± 6.1

Male rats and female rats at proestrus received a 4 μL injection into the left medial forebrain bundle of either vehicle or a dose of 6-OHDA (1 μg or 6 μg) under general anaesthesia. Two weeks later striatal tissue was collected for assessment of dopamine levels using high performance liquid chromatography coupled with electrochemical detection; the hindbrains were also dissected and processed immunocytochemically for tyrosine hydroxylase (TH), the rate-limiting enzyme in dopamine synthesis, as a marker of dopaminergic neurons, which were then counted. The left (lesioned) and right (unlesioned, control) striata and SNc were processed separately. Group means for the lesioned and unlesioned sides were used to calculate the percentage survival of TH immunoreactive (IR) cells. 6-OHDA reduced striata DA levels and TH-IR cell numbers in the SNc in a dose-dependent manner and a sex difference in the severity of the lesion was observed with the dose of 1 μg 6-OHDA. Values represent the mean ± s.e.m. (*n* = 6). Full details in Murray et al. (2003).

⁎ *P* < 0.05 for the effect of 6-OHDA versus vehicle.

⁎⁎ *P* < 0.01 for the effect of 6-OHDA versus vehicle.

# *p* < 0.05 compared with males.
